# Overcoming High Impedance in the Transitional Area of the Distal Great Cardiac Vein during Radiofrequency Catheter Ablation of Ventricular Arrhythmia

**DOI:** 10.3390/jcdd9080264

**Published:** 2022-08-12

**Authors:** Yan-Ru Chen, Yi-Fan Lin, Que Xu, Cheng Zheng, Rui-Lin He, Jin Li, Jia Li, Yue-Chun Li, Jia-Xuan Lin, Jia-Feng Lin

**Affiliations:** Department of Cardiology, The Second Affiliated Hospital and Yuying Children’s Hospital of Wenzhou Medical University, Wenzhou 325000, China

**Keywords:** radiofrequency catheter ablation, ventricular arrhythmia, electrophysiology, distal great cardiac vein, Impedance

## Abstract

(1) Background: Radiofrequency catheter ablation (RFCA) is an essential treatment for ventricular arrhythmia (VA). However, high impedance in the transitional area of the distal great cardiac vein (TAODGCV) often leads to ablation failure. This study aimed to explore the factors influencing impedance and identify effective ways to reduce impedance. (2) Methods: A total of 156 patients with VA arising from the TAODGCV received RFCA therapy at our center from October 2009 to August 2021 and were retrospectively analyzed. Local impedance variation during RFCA was monitored, recorded, and analyzed. (3) Results: The impedance increased from the proximal to distal portions of the TAODGCV and decreased by increasing the saline flow rate at the same site. To overcome high impedance, we implemented the following strategies: (1) Reset the upper limit impedance to 300 Ω and accelerate the saline flow rate to 60 mL/min (effective in 118 of 144 patients); (2) turn off the upper limit impedance (effective in eleven of 21 patients); (3) use high-flow-rate irrigation devices (effective in five of 15 patients); and (4) increase the upper limit temperature (effective in six of ten patients). (4) Conclusions: In the TAODGCV, local impedance is mainly influenced by the target site location and saline flow rate. We concluded several methods to overcome the high impedance and contribute to a successful ablation.

## 1. Introduction

Ventricular arrhythmias (VA), especially premature ventricular contraction (PVC), are common arrhythmias among non-structural heart disease patients [1]. Radiofrequency catheter ablation (RFCA) has been widely used to treat VA [2,3]. A large number of studies have reported catheter ablation of the distal great cardiac vein (DGCV) and its branches (referred to as the transitional area of the DGCV (TAODGCV)) to treat the left ventricular outflow tract (LVOT) epicardial VA. The characteristics of electrocardiography (ECG) and ablation methods for these VAs have been systemically concluded previously [4,5,6,7,8,9,10]. In our clinical practice, high impedance during RFCA in TAODGCV-VA is frequently observed. In extremely high impedance, RFCA energy cannot be delivered, and effective ablation is withdrawn, even though a suitable target site of VAs has been demonstrated. High impedance during RFCA is a significant factor underlying failed ablation in TAODGCV [11,12]. Up to now, no research has comprehensively investigated the effective methods to resolve this problem. This study systemically explored the influencing factors of high impedance in TAODGCV and developed a series of methods to overcome the high impedance.

## 2. Materials and Methods

### 2.1. Study Population

This was a retrospective study of 3276 patients (mean age 49.52 ± 17.56 years) referred to RCFA for VA to our center from October 2009 to August 2021. For 156 of these patients (4.76%), the effective target was in the DGCV, the anterior interventricular vein (AIV), or the summit communicating vein at the top of the left ventricle (Summit-CV), called the TAODGCV-VA. The DGCV is subdivided into DGCV1 (adjacent to the epicardium of the anterolateral wall of the mitral annulus) and DGCV2 (transecting the epicardial left ventricle outflow region bounded by the bifurcation between the left anterior descending artery and the left circumflex artery). The 156 cases included 31 DGCV1 cases (19.87%), 71 DGCV2 cases (45.51%), 35 AIV cases (22.44%), and 19 Summit-CV cases (12.18%). Complete physical and diagnostic examination proved no structural heart diseases, hepatic or renal insufficiency, or coagulation dysfunction in any patient. All patients stopped using anti-arrhythmia medications for at least five half-lives and provided informed written consent before ablation. The hospital’s ethics committee approved the study.

### 2.2. Electrophysiological Examination and Ablation

**Figure 1:**
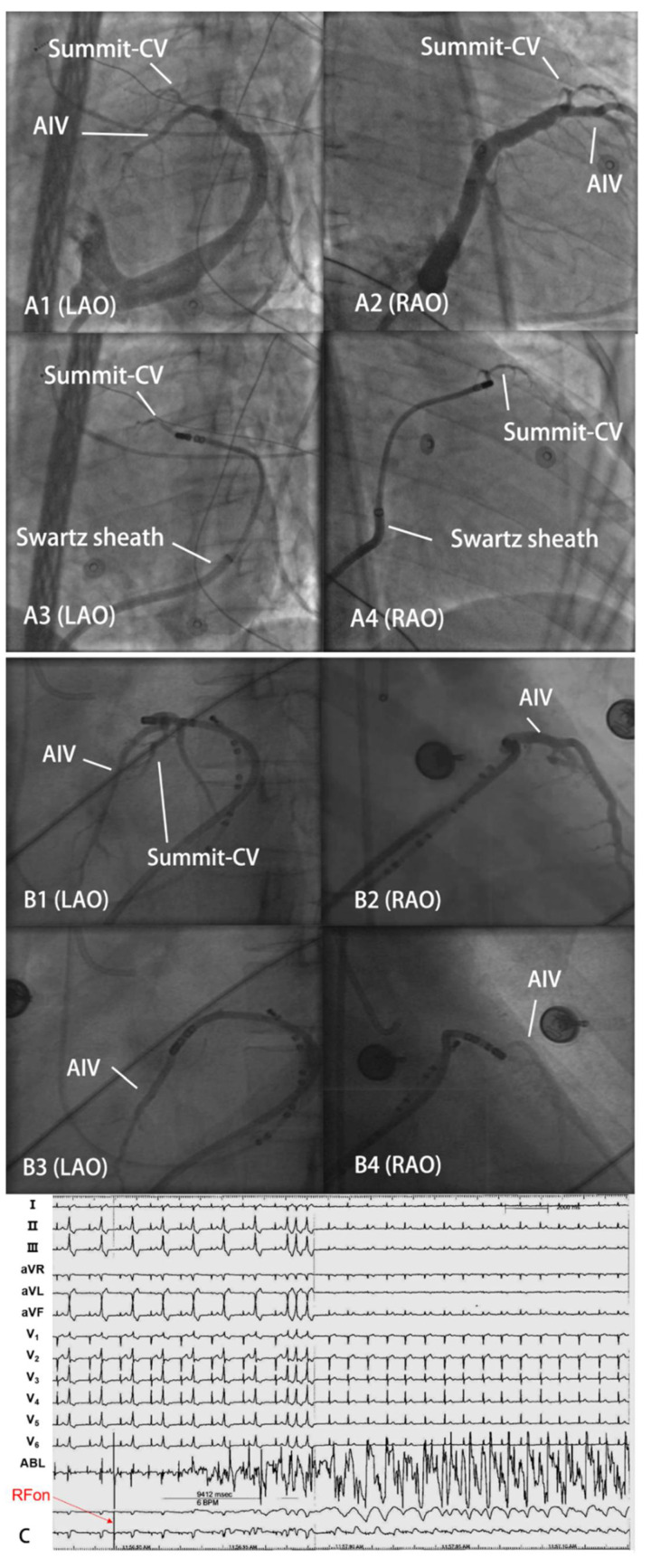
The CVG and electrocardiogram of typical cases. (**A1**–**A4**) shows that the catheter tip reached Summit-CV via the Swartz sheath support approach. (**B1**–**B4**) shows the catheter tip inserted to AIV directly. (**C**) Sample electrocardiogram of a successful ablation of PVC originating from DGCV2. RFon indicates the beginning of radiofrequency energy delivery. The RF energy was delivered at the limit temperature of 43 °C, power of 30 W with a 60 mL/min saline flow rates.

It has been reported that, by non-saline-irrigated catheter, transmural injury was only achieved in 55% of LVOT VAs [13]. Therefore, a saline-irrigated catheter is routinely adopted at our center. Electrophysiological evaluation and catheter ablation were performed as previously described [14]. A 6-F decapolar catheter (4-mm interelectrode spacing) was delivered to DGCV from the jugular vein as distal as possible. Under the guidance of a three-dimensional mapping system (Carto3 or EnSite Velocity) and fluoroscopy, the irrigated catheter (CELSIUS thermo-cool, Biosense Webster or NAVISTAR thermo-cool, Biosense Webster) was applied to map the right ventricular outflow tract, pulmonary sinus, LVOT, and DGCV systemically. If clinical arrhythmias failed to occur spontaneously, intravenous isoproterenol infusion (2–5 mg/min) was administered. The ideal target site of RFCA was determined by activation mapping. When TAODGCV-VAs were considered, coronary venography (CVG) and angiography (CAG) were performed prior to RF application to localize the target site and evaluate the potential risk of coronary artery damage. When the catheter tip failed to reach the target site directly due to the thin lumen of TAODGCV, the Swartz sheath and hydrophilic coated guide wire were adopted [14]. All patients were ablated with the SES system (Stockert EP Shuttle, Biosense, Webster, USA). We preset the limit impedance from 250 Ω to 300 Ω, the temperature was 43 °C, the preset power was 25–35 W, and the saline flow rate was 30–60 mL/min. If the VAs were terminated within 15 s or more and PVCs or non-sustained ventricular tachycardia occurred during ablation at the target site, the additional current was applied for another 60 to 120 s. The target was remapped if the VA was not terminated after 30 s of ablation. Ablation was considered effective when the VA was terminated and could not be induced by intravenous administration of isoproterenol and programmed stimulation. Impedance was measured during the entire process. The original data of local impedance were taken as the average impedance of 5–10 s with relatively stable fluctuation. The CVG and electrocardiogram of typical cases are shown in Figure 1.

### 2.3. Follow-Up and Definition of Outcome

Each patient underwent ECG monitoring for 24–48 h after ablation. 24-h Holter monitoring was performed at 1, 3, 6, and 12 months after ablation to evaluate the long-term effects.

Acute success was defined as the complete elimination of spontaneous or inducible VA during the procedure and no VA recurrence within 24 h postoperatively. Long-term success was defined as the PVC burden decreasing by at least 80% of that of prior ablation for three months [15].

### 2.4. Statistical Analysis

Continuous variables were expressed as mean ± standard deviation. For discontinuous variables, the percentages were reported. The two-sample t-test was used to compare the continuous variables, and the χ^2^ test or Fisher’s exact test was used to compare the discontinuous variable groups. A *p* < 0.05 was considered significant. Statistical analyses were performed using IBM SPSS Statistics Version 26.

## 3. Results

### 3.1. Patient Characteristics

One hundred and fifty-six patients with TAODGCV-VA who underwent RFCA in TAODGCV at our center were enrolled in this study and retrospectively analyzed. There were 141 cases of palpitation (90.38%), 106 cases of chest tightness (67.95%), nine cases of chest pain (5.77%), and four cases of amaurosis (2.56%). The quality of life of all cases was severely affected; these symptoms could not be alleviated by at least one antiarrhythmic mediation. The characteristics of the patients are shown in Supplementary Table S1.

### 3.2. Ablation Characteristics

After RFCA therapy, a total of 134 patients achieved acute success. The reasons for failure included: (1) the catheter tip failed to reach the target site because of the thin lumen of TAODGCV (seven cases); (2) the distance between the target and the left anterior descending coronary artery (LAD) was less than 5 mm, and ablation is forbidden (five cases); (3) pacing could only capture the atrium, suggesting that the DGCV was located more on the side of the atrium, and ablation was ineffective (five cases); (4) extremely high impedance that could not overcome by each method (four cases); and (5) unknown reason; in one case, activation mapping showed the earliest local ventricular activation in the Summit-CV preceding QRS onset by 36 ms; however, ablation still failed. The characteristics of mapping and ablation are shown in Supplementary Table S2.

### 3.3. Factors Influencing Impedance

**Figure 2:**
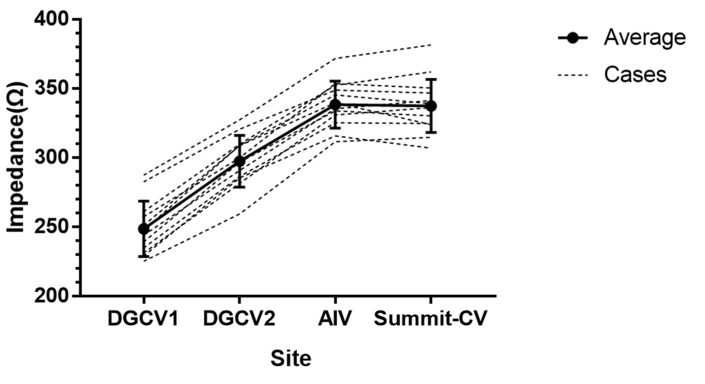
The impedance in different parts of the TAODGCV. The above impedance values were measured at the initial state (saline flow rate was 2 mL/min).

**Figure 3:**
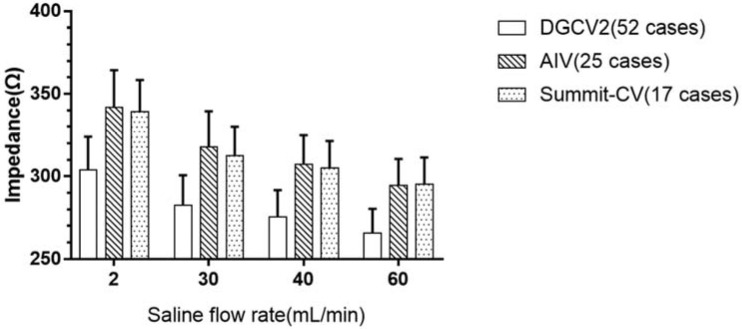
The average impedance under the different saline flow rates. DGCV2 = distal great cardiac vein 2; AIV = anterior interventricular vein; Summit-CV = summit communicating vein at the top of the left ventricle.

Among the 156 cases, we reached the target site in TAODGCV in 149 patients, while target sites were not achieved by the catheter tip in seven patients due to the tortuous DGCV, the acute angle between the DGCV and the AIV, or the thin lumen of Summit-CV. We found the impedance gradually increased in these 149 patients from proximal to distal TAODGCV (DGCV1 < DGCV2 < AIV/Summit-CV). The impedance decreased significantly during the pre-RF time with pre-flush (saline flow rate was 30 mL/min) (Table 1). TAODGCV could be accessed entirely by the catheter tip in only 12 cases. In each case, the impedance increased from the proximal to the distal portion of the TAODGCV (DGCV1 < DGCV2 < AIV/Summit-CV) (Figure 2). A total of 94 patients (except those originating from DGCV1) had an intraoperative saline flow rate of 60 mL/min or more. The average impedance measured in these patients is shown in Figure 3. At the same site, the impedance decreased with the increase in the saline flow rate.

### 3.4. Ways to Overcome High Impedance

**Figure 4:**
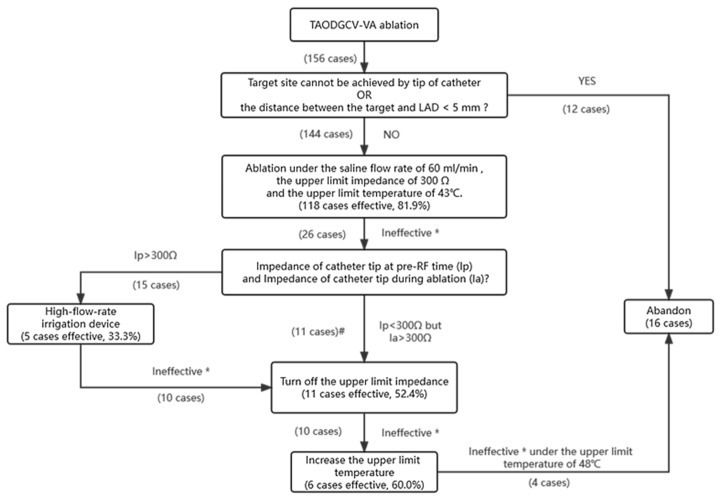
The ablation process of patients with TAODGCV-VA. TAODGCV-VA = =ventricular arrhythmia originating from the transitional area of the distal great cardiac vein; LAD = left anterior descending coronary artery. * Ineffective means does not meet the standard of effective ablation (VA is terminated and cannot be induced by intravenous administration of isoproterenol and programmed stimulation, which usually requires energy delivery >20 W and lasting >30 s). # Ineffective because the spontaneous cut-off caused by the sharp impedance increase during ablation.

Excluding cases where the catheter tip could not achieve the target site (seven cases) and those reaching the target but the distance between the target and LAD was <5 mm (five cases), the remaining 144 cases underwent ablation. However, effective ablation was achieved in only three patients (all from DGCV1) at the preset saline flow rate of 30 mL/min and the upper limit impedance of 250 Ω. Ablation was forbidden or cut-off in 141 patients because of the initial high impedance of the TAODGCV or the rapid increase in impedance during ablation. We increased the saline flow rate to reduce the impedance and adjust the upper impedance limit as high as possible. Effective ablation was achieved in most patients (118 of 141 cases, 81.9%) under the saline flow rate of 60 mL/min and the upper limit impedance of 300 Ω (both were the maximal values that can be provided by the ablation instrument). Ablation was forbidden in 15 cases (15/26, 57.7%) due to the impedance at the pre-RF time higher than the maximal limit impedance of 300 Ω and cut-off in eleven patients (11/26, 42.3%) due to high impedance (>300 Ω) during ablation. To further reduce the pre-RF impedance, a high-flow-rate irrigation device (flow rate of 120 mL/min) was adopted in 15 cases. However, effective ablation was only achieved in five patients (5/15, 33.3%). In the remaining ten patients and eleven patients whose pre-RF time impedances were lower than 300 Ω (a total of 21 cases), effective energy delivery was stopped by rapid increase impedance, and we turned off the upper limit impedance. This way, energy delivery could continue, and effective ablation was achieved in eleven patients (11/21, 52.4%). After adopting the above-mentioned methods, the energy delivery could not reach 20 W in ten cases, and PVC disappeared but recurred immediately after ablation. As it was impossible to reduce the impedance further, we had to increase the upper limit temperature to increase the output power. By upregulating the upper limit temperature to 45 °C in four cases, 46 °C in one case, and 48 °C in another case, stable energy ranging from 20 to 25 W could be delivered, and successful elimination of PVC was reached. Ablation was abandoned in four cases after all attempts. The procedure of ablation treatment is shown in Figure 4, and the details of the ablation strategies used are shown in Table 2. Most cases (140/144, 97.22%) overcame high impedance using these ablation strategies.

### 3.5. Complications

As shown in Table 3, there were ten untreated coronary vein dissection cases (including five transient contrast staining cases and five persistent contrast staining cases); two cases developed coronary vein rupture with pericardial effusion and both improved after pericardiocentesis. Acute coronary artery injury occurred in three cases, and two of them with coronary artery spasm (35% and 40% of LAD stenosis, respectively, without evident symptoms, with improvement after intracoronary injection of nitroglycerin). One case developed severe chest pain after ablation for about 9 s (coronary angiography showed 50% of LAD stenosis) and disappeared after 15 min. Delayed pericardial effusion occurred 22 days after symptomatic treatment and improved after pericardiocentesis (with 540 mL of yellowish effusion) and corticosteroid therapy. No steam-pops occurred in this study. In addition, there was no significant correlation between the complications and the strategies we adopted to overcome high impedance. The details of the cases who experienced complications is shown in Supplementary Table S3.

### 3.6. Follow-Up Results

The outcomes of all 156 patients are shown in Supplementary Figure S1; 140 cases achieved effective ablation and 134 (85.90%) achieved acute success. In two of the six failure cases, VAs disappeared several seconds after ablation but reappeared immediately, and VAs disappeared after follow-up for 3 months to 1.5 years. During follow-up, recurrence occurred in three cases (2.24%) and all recurrences occurred within three months after ablation (Supplementary Figure S2). Three patients with coronary artery injury and spasms underwent CT angiography (CTA) 3 to 6 months after the operation, and no severe complications were found, except for one case of delayed pericardial effusion.

## 4. Discussion

### 4.1. Major Findings

**Figure 5:**
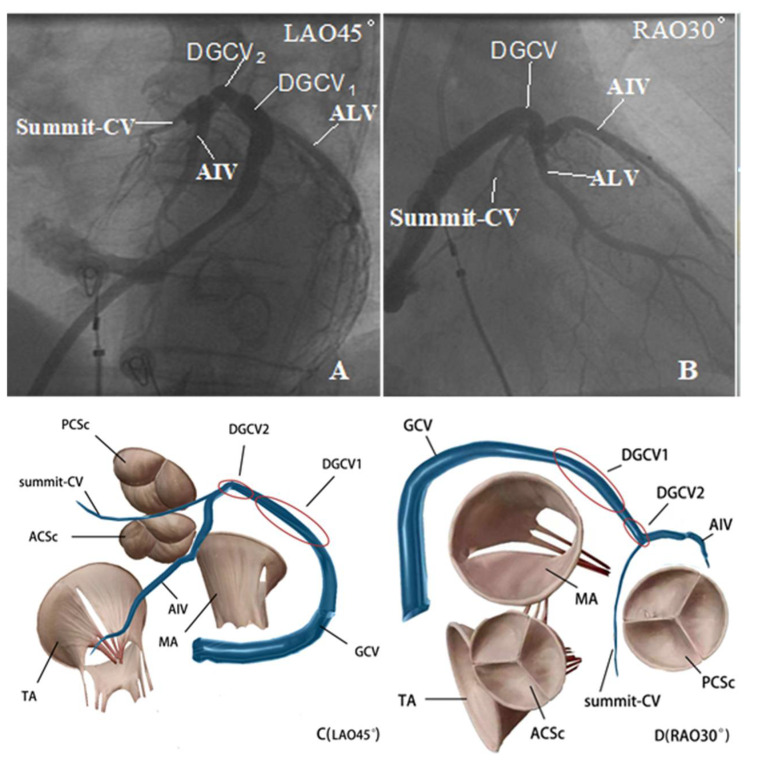
The X-ray imaging and anatomy of TAODGCV. (**A**,**B**) are the X-ray imaging of TAODGCV. (**C**,**D**) are anatomical diagrams of TAODGCV. The red ovals indicate the range of DGCV1 and DGCV2 defined in this study. TAODGCV = transitional area of the distal great cardiac vein; DGCV1 = distal great cardiac vein 1; DGCV2 = distal great cardiac vein 2; GCV = great cardiac vein; AIV = anterior interventricular vein; Summit-CV = summit communicating vein at the top of the left ventricle; TA = tricuspid annulus; MA = mitral annulus; ALV = anterior lateral vein; LAO = left anterior oblique position; RAO = right anterior oblique position.

We investigated the impedance of the catheter tip during the RFCA of TAODGCV VAs and observed that impedance during the procedure was affected by anatomical structure (distal or proximal TAODGCV) and saline flow rate of the catheter tip. Anatomically, the diameter of GCV decreases from proximal to distal (Figure 5). Van de Veire et al. demonstrated that the diameter of the proximal GCV was 7.2 ± 1.4 mm on average, while the distal was 4.9 ± 1.1 mm) [16]. A study using contrast-computed tomography showed a thin lumen of AIV with a mean ostial diameter of 4.46 mm [17]. Summit-CV is the thinnest part of the TAODGCV, and Komatsu et al. reported that due to the very thin lumen of Summit-CV in some patients, only the 2-F mapping catheter could access this site [18]. The law of resistance states that the larger the cross-sectional area, the lower the impedance (R = ρl/S, where R is the impedance, ρ is the resistivity, l is the length, and S is the cross-sectional area). This equation explains the phenomenon we observed where AIV and Summit-CV had higher impedance while DGCV1 and DGCV2 had lower impedance as the AIV and summit-CV were relatively much thinner than DGCV1 and DGCV2. In addition, there was no significant fat between Summit-CV and epicardial surface, while other parts of TAODGCV run through or are on the thick overlying epicardial fat pads that contribute to high impedance [19]. This phenomenon explains why Summit-CV had the smallest diameter, but the impedance was similar to AIV.

We also found that the impedance was negatively correlated with the saline flow rate. We believe that the law of resistance could explain this finding. First, due to the high elasticity of the vessel wall, with the increase in saline flow rate, the vessel lumen is filled, and the cross-sectional area of blood vessels is comparably increased. Second, the resistivity of saline is significantly lower than that of blood (about 0.8 Ω·m vs. 10,000 Ω·m). These factors resulted in the decrease of the impedance by increasing the saline flow rate. In our experience, we recommend an initial preset saline flow rate of 60 mL/min in TAODGCV-VA patients because most cases require this rate. However, the cooling effect of high flow rate can impede transmural lesion formation, which may lead to an inadequate ablation. Fortunately, the patients who suffered inadequate ablation usually had the characteristic of the transient disappearance of VA during ablation (VA disappeared when discharging, but reappeared soon after energy delivery cut-off). At this time, longer RF duration and higher power are required. Additionally, more RF lesions need to be created if necessary. Therefore, more ablation strategies (even increase the upper limit temperature) were adopted in these patients to enhance the effect of ablation. In a word, the advantages of high flow rate in DGCV outweigh the disadvantages, and its weakening effect on ablation can be overcome.

Impedance decreases during RFCA are specific markers of target site heating. The appropriate impedance decrease is critical for effective ablation. Excessive decrease in impedance will increase the incidence of complications, while a decrease in impedance that is too low indicates ineffective treatment [20,21]. The impedance changes rapidly when emergencies such as steam pops occur. Due to the anatomical structure of the TAODGCV, the high impedance at the pre-RF time and the sharp impedance increase during ablation are common; this phenomenon restrains the energy delivery and contributes to a failed ablation. Increasing the upper limit impedance can overcome this problem. In practice, we recommend presetting an initial upper limit impedance of 300 Ω for ablation in TAODGCV-VA. However, an impedance greater than 300 Ω is not rare in TAODGCV, and a previous study found that some patients failed ablation because of the high impedance [18]. For patients with an impedance at a pre-RF time of less than 300 Ω (whereas impedance sharply increases to more than 300 Ω during ablation), we chose to abandon impedance detection to avoid the cut-off of energy delivery. Though impedance detection guarantees safe ablation, the protective effect of impedance detection is substantially weakened because of the enormous fluctuation of impedance in these patients. Furthermore, we paid more attention to the real-time monitoring of the catheter tip temperature to ensure the safety of the ablation. Ablation should be stopped immediately when the temperature rises sharply in a short period. In addition, repetitive CAG and CVG were performed during RFCA, which further reduced the potential risk of coronary artery and vein damage. We turned off the upper impedance limit in 21 cases, and none experienced complications during energy delivery (two cases experienced coronary vein dissection caused by catheter manipulation). However, turning off the upper limit impedance did not help reduce the impedance and could not solve the problem of low ablation power output in patients with an impedance greater than 300 Ω at the pre-RF time (at constant voltage, the power and impedance are negatively correlated [P = U^2^/R, where P is power, U is voltage, and R is impedance]). For these patients, we recommend reducing the impedance by using a high-flow-rate irrigation device to increase the power first, rather than turning off the upper limit impedance immediately.

Studies have shown that excessive temperature increases the risk of complications caused by injury to a nearby anatomical structure [22,23]. When ablation occurs with a temperature limit of 45 °C, the risk can be reduced while ablation is effective [24]. In the present study, we set the temperature to 43 °C to ensure safety. However, for patients whose energy delivery remained too low after using the methods above-mentioned (effective ablation requires more than 20 W of energy delivery), we can turn up the upper limit temperature as a last resort. One patient could not be ablated until the temperature was raised to 48 °C, and the follow-up result showed that the ablation was safe and effective. Four patients who finally failed ablation also underwent ablation at an upper limit temperature of 48 °C, and none experienced complications during energy delivery (one case experienced coronary vein dissection caused by catheter insertion). A study reported that the catheter tip temperature of 16 patients reached more than 50 °C during ablation treatment without complications [24]. We consider ablation at the upper limit temperature of 48 °C to be safe; however, this method should only be used after all other efforts are ineffective, and the temperature must be gradually increased to minimize risk.

### 4.2. Different Ablation Strategies in the Various Parts of the TAODGCV

The vessels in various parts of the TAODGCV differ in terms of course, thickness, and adjacency; therefore, the ablation strategies also differ. DGCV1 and DGCV2 are in the main branch of the cardiac vein and have relatively low impedance. Therefore, the direct arrival rate and ablation rate were higher than the AIV and Summit-CV. All patients with effective targeting at DGCV1 were ablated effectively by resetting the upper limit impedance and increasing the saline flow rate. Only 13 cases originating from DGCV2 required further treatment.

The AIV and Summit-CV had many bends, large angulations, and small diameters as the secondary branches. Most required Swartz sheath support, CVG guidance, and additional treatments to reduce impedance. Furthermore, we found that the anatomical variation of AIV is common and is related to the LAD in two ways: (1) the AIV descends along the anterior interventricular sulcus in parallel on the left side of LAD; at this point, the distance between AIV and LAD is about 10 mm, and ablation is safe; and (2) the AIV crosses from the left side of the LAD to the right. At this point, the relationship between the target and the LAD should be clarified to ensure safe ablation (<5 mm is dangerous for ablation).

### 4.3. Safety and Complications of Ablation

Coronary vein dissection was the most common complication (6.41%). In this study, coronary vein dissection was defined as contrast staining observed by CVG during RFCA, which included transient (within 30 s) and persistent contrast staining. Transient contrast staining indicates minor dissection with contrast diffused quickly, while persistent contrast staining means relative larger dissection during RFCA, which needs more attention. Many studies have revealed that venous dissection could be resealed spontaneously due to the repair function of endothelium, and the ability of self-healing supports conservative therapy without further intervention [25,26,27]. Furthermore, the direction of blood flow in the vein is opposite to the direction of dissection caused by catheter insertion, so the blood flow can compress the dissection and promote recovery. In all ten coronary vein dissection patients, there was no adverse events occurrence in-hospital and out-hospital. Thus, we consider that coronary vein dissection is actually a complication with a high occurrence rate, however, it did not result in catastrophic outcome.

CVG was performed by catheter tip prior and post each energy delivery and no new onset coronary venous injury (including transient contrast staining, persistent contrast staining, and coronary vein rupture and pericardial effusion) was observed. Thus, it is reasonable to believe that coronary venous injury was caused by forced catheter insertion to the distal cardiac vein (mechanical manipulation) but not ablation itself. The insertion of the catheter needs to go through many anatomical obstacles including venous valves (Thebesian valve and Vieussens valve), deflections of the great cardiac vein, acute angle between DGCV and AIV, the thin lumen of TAODGCV, and so on. Thus, coronary venous injury cannot be completely avoided during catheter manipulation, which contributes to the high complication rate.

The patients with coronary artery injury or spasms underwent CTA 3–6 months after surgery, and no other severe complications were found. In addition, in three cases, though the distance between the AIV ablation target and LAD was >5 mm, coronary artery injury or spasticity still occurred. Furthermore, no steam-pops occurred in this study, which may have been caused by effective cooling of the high saline flow rates.

Above all, if the insertion of the catheter is gentle, it is safe and effective to perform the ablation of VA in various parts of the TAODGCV by using fine mapping, timely CVG, and CAG to judge the anatomical direction of the coronary artery and vein and the adjacent relationship with the ablation target.

### 4.4. Study Limitations

(1) Because this study was a single-center non-randomized case-control study, multicenter randomized clinical trials are needed to validate our findings. (2) Most of the patients had not been examined using intracardiac ultrasound, which may have affected the location of effective targets. Nevertheless, CVG and CAG are superior to intracardiac ultrasound for target location and delineating clear neighbor relationships with the coronary arteries. (3) Except for three patients with coronary artery injury or spasm during the operation, most patients did not undergo CAG or CTA during follow-up to evaluate the long-term effect of ablation on adjacent coronary arteries; we are currently working to rectify this limitation. (4) All patients were ablated with the SES system in this study, and our center replaced a new generation of ablation instruments in 2021. We found that the ablation efficiency of different ablation instruments in the high impedance environment is different, which may have an impact on the practice of the above ablation strategies.

## 5. Conclusions

In the TAODGCV, local impedance is influenced by the target site location (distal or proximal DGCV) and saline flow rate. When high impedance occurred, raising or turning off the upper limit impedance and increasing the saline flow rate effectively overcame the high impedance. Turning up the upper limit temperature may work for patients whose energy delivery remained too low after using the above methods.

## Figures and Tables

**Table 1:** The average impedance in different parts of TAODGCV at the initial state and the pre-RF time (Ω, $\bar{x}\pm S$).

Target Site Location	DGCV1 (n = 30)		DGCV2 (n = 70)		AIV (n = 32)		Summit-CV (n = 17)	
	Initial State *	Pre-RF Time ^#^	Initial State	Pre-RF Time	Initial State	Pre-RF Time	Initial State	Pre-RF Time
Impedance	250.8 ± 21.2	221.6 ± 15.8 ^a^	300.4 ± 17.3 ^b^	265.7 ± 23.1 ^a, b^	341.6 ± 20.6 ^b, c^	292.8 ± 21.3 ^a, b, c^	338.5 ± 21.7 ^b, c^	296.4 ± 17.8 ^a, b, c^

* In the initial state, the saline flow rate was 2 mL/min. ^#^ In the pre-RF time, the saline flow rate was 30 mL/min. ^a^ Compared with the initial state of the same target site location, *p* < 0.01; ^b^ Compared with DGCV1 in the same state, *p* < 0.01; ^c^ Compared with DGCV2 in the same state, *p* < 0.01.

**Table 2:** The details of the ablation strategies used in different target site locations (cases).

**Target Site Location (n = 144)**	**Saline Flow Rate**			**Limit Impedance**		
	**30 mL/min**	**40 mL/min**	**60 mL/min**	**250 Ω**	**300 Ω**	**Turn Off**
DGCV1 (n = 30)	6	16	8	3	27	0
DGCV2 (n = 68)	2	14	52	0	58	10 (5 effective)
AIV (n = 29)	0	4	25	0	22	7 (4 effective)
Summit-CV (n = 17)	0	0	17	0	13	4 (2 effective)
Sum	8	34	102	3	120	21 (11 effective)
**Target Site Location (n = 144)**	**High-Flow-Rate Irrigation Devices**		**Increase the Upper Limit Temperature**		**Abandon**	
DGCV1 (n = 30)	0		0		0	
DGCV2 (n = 68)	6 (3 effective)		5 (3 effective)		2	
AIV (n = 29)	4 (2 effective)		3 (1 effective)		2	
Summit-CV (n = 17)	2 (none effective)		2 (2 effective)		0	
Sum	15 (5 effective)		10 (6 effective)		4	

**Table 3:** The complications of radiofrequency catheter ablation in TAODGCV.

Complication	All Cases (n = 156)	Turn off the Upper Limit Impedance (n = 21)	High-Flow-Rate Irrigation Devices (n = 15)	Increase the Upper Limit Temperature (n = 10)
Coronary vein dissection *				
Transient contrast staining	5 (3.21%)	1 (4.76%)	0	1 (10.0%)
Persistent contrast staining	5 (3.21%)	1 (4.76%)	1 (6.67%)	0
Coronary vein rupture	2 (1.28%) ^a, b^	0	0	0
Acute pericardial effusion	2 (1.28%) ^a, b^	0	0	0
Delayed pericardial effusion	1 (0.64%)	0	0	0
Coronary artery injury	3 (1.92%) ^c, d^	0	0	0
Coronary artery spasm	2 (1.27%) ^c, d^	0	0	0
Death	0	0	0	0
Sum	16 (10.26%)	2 (9.52%)	1 (6.67%)	1 (10.0%)

^a^, ^b^, ^c^ and ^d^ represent the same patient. * Coronary vein dissection included transient (within 30 s) and persistent contrast staining.

## Data Availability

Data are available on reasonable request to the corresponding author.

## Supplementary Materials

The following supporting information can be downloaded at: https://www.mdpi.com/article/10.3390/jcdd9080264/s1, Table S1: The patient characteristics; Table S2: The characteristics of mapping and ablation; Table S3: The details of the cases who experienced complications; Figure S1. Follow-up outcomes of all 156 patients; Figure S2. A Kaplan–Meier curve of patients who achieved acute success.
