# Supplementary material for: Overcoming High Impedance in the Transitional Area of the Distal Great Cardiac Vein during Radiofrequency Catheter Ablation of Ventricular Arrhythmia
Source: J Cardiovasc Dev Dis. 2022 Aug 12;9(8):264. doi: 10.3390/jcdd9080264 (PMC9410161; doi:10.3390/jcdd9080264)
Supplement: Supplementary file 1 [file jcdd-09-00264-s001.zip › jcdd-1815018-supplementary.pdf]

Supplementary Table S1. patient characteristics.

	Patients (n = 156)
Sex(Male %)	96(61.53%)
Age(year)	57.22±15.68
Total number of PVCs (times/24h) #	22472±9647
Course of disease (year)	2.83±1.96
Complicated with hypertension	18(11.54%)
LVEDd(mm)	48.33±5.82
LVEF (%)	65.72±5.88
Complicated with left ventricular hypertrophy	23(14.74%)
NSVT	25(16.03%)
SVT	11(7.05%)
PVCs	110(70.51%)

#Dynamic electrocardiogram was performed in 144 cases. PVC indicates premature ventricular contraction; LVEF, left ventricular ejection fraction; LVEDd, left ventricular end-diastolic dimension; NSVT, non-sustained ventricular tachycardia; SVT, sustained ventricular tachycardia.

Supplementary Table S2. the characteristics of mapping and ablation.

	Patients (n=149)
V-QRS (ms)	-34.96±4.82
Ventricular capture (case,%)	132(88.59%)
Pace march leads	11.38±1.28
Procedure time(min)	62.58±11.23
Operation in CS (min)	25.91±4.53
RF duration (s)	146.28±47.03
No. of RF lesions	1.72±0.63
Fluoroscopy time (min)	8.62±3.86

Supplementary Table S3. Details of cases who experienced complications.

Patients	Complication	Age, year	Sex	Origin	Power, W	Upper limit impedance,Ω	Saline flow rates, ml/min	Upper limit temperature, ℃
1	Transient contrast staining	62	F	DGCV1	30	300	40	43
2	Transient contrast staining	54	M	DGCV1	25	300	40	43
3	Transient contrast staining	55	F	DGCV1	30	300	60	43
4	Transient contrast staining	62	F	DGCV2	30	300	60	43
5	Transient contrast staining	34	F	Summit-CV	45	Turn off	60	45
6	Persistent contrast staining	65	M	DGCV2	25	300	60	43
7	Persistent contrast staining	70	M	DGCV2	35	300	60	43
8	Persistent contrast staining	46	M	AIV	25	300	60	43
9	Persistent contrast staining	69	F	AIV	30	Turn off	120	43
10	Persistent contrast staining	82	M	Summit-CV	25	300	60	43
11	Coronary vein rupture/ Acute pericardial effusion	71	F	DGCV1	30	300	60	43
12	Coronary vein rupture/ Acute pericardial effusion	81	M	AIV	25	300	60	43
13	Delayed pericardial effusion	56	F	Summit-CV	25	300	60	43
14	Coronary artery injury	46	M	DGCV2	30	300	60	43
15	Coronary artery injury/ Coronary artery spasm	38	M	DGCV2	35	300	60	43
16	Coronary artery injury/ Coronary artery spasm	44	F	AIV	25	300	60	43

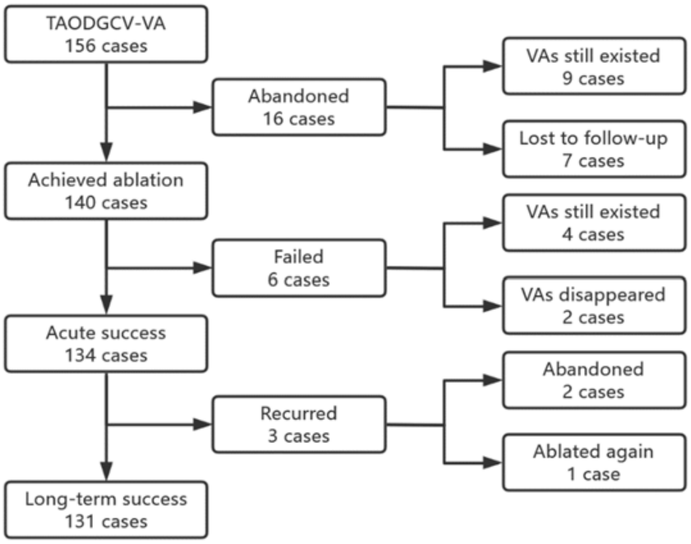

Supplementary Figure S1. Follow-up outcomes of all 156 patients. TAODGCV = Transitional area of the distal great cardiac vein; VA = Ventricular arrhythmia. Achieve ablation was defined as energy release reaching 20–25 W and lasting 30 seconds. Acute success was defined as ventricular arrhythmia no longer being induced by electrical stimulation or intravenous infusion of isoproterenol after ablation, and ventricular arrhythmia was eliminated within 24 hours postoperatively per electrocardiography monitoring. Long-term success was defined as premature ventricular contractions decreasing by more than 80% for three months after ablation, significantly improving symptoms. Recurrence was defined as premature ventricular contractions PVC burden decreasing by at least 80% of that of prior ablation for three months [15].

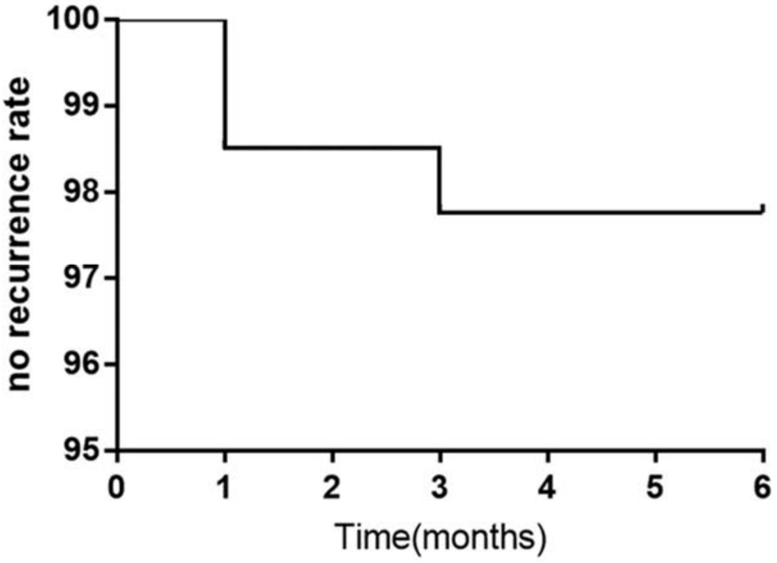

Supplementary Figure S2. a Kaplan-Meier curve of patients who achieved acute success.

All recurrences occurred within three months after ablation.
